# Effects of arbuscular mycorrhizal inoculation on the growth, photosynthesis and antioxidant enzymatic activity of *Euonymus maackii* Rupr. under gradient water deficit levels

**DOI:** 10.1371/journal.pone.0259959

**Published:** 2021-11-23

**Authors:** Na Wu, Zhen Li, Sen Meng, Fei Wu

**Affiliations:** 1 Institute of Applied Biotechnology, School of Life Science, Shanxi Datong University, Datong, Shanxi, China; 2 State Key Laboratory of Tree Genetics and Breeding, Research Institute of Tropical Forestry, Chinese Academy of Forestry, Guangdong, China; 3 2011 Collaborative Innovation Center of Jiangxi Typical Trees Cultivation and Utilization, College of Forestry, Jiangxi Agricultural University, Nanchang, Jiangxi, China; Universidade de Coimbra, PORTUGAL

## Abstract

The role of arbuscular mycorrhizal (AM) fungus (*Rhizophagus intraradices*) in the amelioration of the water deficit-mediated negative influence on the growth, photosynthesis, and antioxidant system in *Euonymus maackii* Rupr. was examined. *E*. *maackii* seedlings were subjected to 5 water deficit levels, soil water contents of 20%, 40%, 60%, 80% and 100% field capacity (FC), and 2 inoculation treatments, with and without AM inoculation. The water deficit increasingly limited the seedling height, biomass accumulation in shoots and roots, chlorophyll content, gas exchange and chlorophyll fluorescence parameters with an increasing water deficit level. In addition, water deficit stimulated the activities of antioxidant enzymes, including superoxide dismutase (SOD), peroxidase (POD) and catalase (CAT), in both shoots and roots, except under 20% FC conditions. *E*. *maackii* seedlings under all water deficit conditions formed symbiosis well with AM fungi, which significantly ameliorated the drought-mediated negative effect, especially under 40% and 60% FC conditions. Under 40% to 80% FC conditions, AM formation improved seedling growth and photosynthesis by significantly enhancing the biomass accumulation, chlorophyll content and assimilation. Mycorrhizal seedlings showed better tolerance and less sensitivity to a water deficit, reflected in the lower SOD activities of shoots and roots and CAT activity of shoots under 40% and 60% FC conditions. Downregulation of the antioxidant system in mycorrhizal seedlings suggested better maintenance of redox homeostasis and protection of metabolism, including biomass accumulation and assimilation. All the results advocated the positive role of *R*. *intraradices* inoculation in *E*. *maackii* against a water deficit, especially under 40% FC, which suggested the distinct AM performance in drought tolerance and the potential role of the combination of *E*. *maackii*-AM fungi in ecological restoration in arid regions.

## 1. Introduction

Continuous economic activities have induced severe environmental concerns, such as soil salinization, soil erosion, and a water deficit, which have seriously limited plant growth [1, 2]. Water deficit is one of the major limiting factors on agricultural and forest yield [3], and it has emerged as an alarming global stress factor, causing the excessive loss of agricultural production and ecosystem damage. In arid and semiarid regions, the incidence of water deficit episodes has increased in recent decades [4]. Even in nonarid regions, seasonal drought cannot be avoided [5].

*Euonymus maackii* Rupr. is an important ecological restoration tree species and is widely cultivated in northwestern China. *E*. *maackii* is economically important as a material for biodiesel [6]. However, it is a highly water-consuming species and is severely limited by water deficit episodes. Drought causes severe damage to plant growth by influencing the antioxidant enzymatic activity, photosynthetic capacity, and mineral nutrient uptake [7–9]. A water deficit induces stomatal closure and photosystems I and II corruption, resulting in limited access to CO_2_. A drought-mediated decrease in growth and biomass accumulation results from a decline in photosynthesis [10].

When a water deficit occurs, reactive oxygen species excessively accumulate in plant tissue, causing serious oxidative damage, structural and functional damage to the integrity of cellular organelles, and upregulation of antioxidant enzymatic activities, such as superoxide dismutase (SOD), peroxidase (POD) and catalase (CAT) [10, 11]. The antioxidant system is considered to eliminate the excess accumulation of reactive oxygen species to protect plant metabolism. The negative effect of water deficit stress on plants depends on the severity and duration of the stress [7, 12].

Arbuscular mycorrhizal (AM) symbiosis occurs in almost all terrestrial ecosystems and has been widely reported to form mutually beneficial relationships with most terrestrial plants [13]. AM symbiosis improved the plant capacity to tolerate a water deficit in a variety of ways, such as enhancing water and mineral nutrient uptake, improving photosynthesis, regulating the hormonal status of plants and upregulating the antioxidant system as well as gene expression of stress-responsive genes [13–17]. AM-mediated regulation of plant morphological and physio-chemical processes, such as improvement in water uptake and mineral assimilation, is reflected in better performance in photosynthesis and biomass accumulation [4, 5, 15, 18].

To investigate the effect of AM inoculation treatment on *E*. *maackii* when subjected to different water deficit levels, we set this study and hypothesize that 1) water deficit would limit the growth, photosynthesis, and stimulation of the antioxidant system of *E*. *maackii*; 2) the negative effect of drought would increase with an increase in the water deficit level; and 3) AM inoculation treatment would enhance the ability of the plant to cope with water deficit. Furthermore, different attributes between moderate and severe water deficits result in different responses of seedlings, which could ultimately result in distinct AM performance in drought tolerance.

## 2. Methods

### 2.1 AM inoculum

*Rhizophagus intraradices* [Schenck & Smith (BGC BJ09)] provided by the Beijing Academy of Agriculture and Forestry Science was used for AM inoculation in this study. The AM inoculum was expanding propagated with *Trifolium* *repens* and consisted of AM spores (approximately 50 spores per gram of inoculum), mycelia, root fragments and soil.

### 2.2 Plants and soil treatment

Plump and healthy seeds of *E*. *maackii*, provided by the Shaanxi Provincial Forestry Technical Extension Station, were selected and disinfected in 0.5% KMnO_4_ solution for 20 min, washed 3 times with sterile water, and soaked in sterile water for 24 h. Seeds were transferred to a salver covered with wet gauze and kept at 25°C under 12 h of light in an illuminated incubator for rapid germination. After the seeds germinated and grew to 1 cm, the seedling pot (47 cm × 33 cm, 66 holes) filled with sterilized vermiculite was used for further cultivation. One germinated seed was put in one hole, and the seedling pot was placed at 25°C and 12 h of light in an illumination incubator. The seeds were watered every morning (20 ml/hole) for 1 month, and fully grown seedlings with similar growth were transplanted to the pots described below.

Topsoil of 5–20 cm in depth was collected from a field of *E*. *maackii*. The soil, sieved through a 2 mm sieve, was mixed with fine washed sand (v: v = 1: 1). The mixed substrate was then autoclaved at 0.11 MPa and 121°C for 20 min for further pot experiments. The topsoil physicochemical properties were measured as described by Li [15] and as follows: soil organic carbon, 16.37 g∙kg^-1^; available K, 111.08 mg∙kg^-1^; available N, 33.36 mg∙kg^-1^; available P, 10.25 mg∙kg^-1^; and pH value (soil: water = 1:5), 7.9. The field capacity (FC) of the mixed substrate was 22.50%. Compared with soil, the nutrition of fine washed sand used was very low and could be ignored.

### 2.3 Experimental design

The experimental design included 2 factors: inoculation treatment (inoculated with *R*. *intraradice*s or autoclaved inoculum) and gradient of the water deficit (20%, 40%, 60%, 80% and 100% FC). Seedlings of similar growth were planted in 1 l plastic pots filled with 1 kg of preprocessed substrate and grown in an artificial climate greenhouse at 25–30°C with 12 h of light per day and a stable humidity of 50%. Fifteen replications of each treatment were used in this experiment, totaling 150 seedlings (2×5×15). Half of the pots were inoculated with 10 g of AM inoculum, and the remaining pots were nonmycorrhizal controls and inoculated with 10 g of autoclaved inoculum. All the seedlings were well watered for the first 3 months with sterile water, and 150 ml of Hoagland’s complete nutrient solution [19] was added every 2 weeks. After 3 months, pots subjected to 20%, 40%, 60%, and 80% FC treatments remained unwatered until their soil water content reached the desired FC levels. The remaining pots were subjected 100% FC treatment and were kept well-watered [15]. Pots were arranged in a randomized complete block design. All pots were kept at a stable water content for 30 d and then harvested. All pots were weighed and watered every day at 17:00 to maintain the water content at the desired FC levels. All the indexes were measured with 6 randomly selected seedlings of each treatment.

### 2.4 AM colonization rate and AM dependency measurement

The roots were cleared and stained as described by Phillip and Hayman [20], and the AM colonization rate was detected and measured using optical microscope. At harvest, the seedlings with substrate on the roots were moved gently from the pot. The roots were washed carefully with flowing tap water to remove the substrate and then rinsed 3 times with distilled water. Fine roots (< 2 mm) were cut into 1-cm-long segments and bleached in 5% KOH at 90°C in a water bath. Then, the cleared root segments were acidified in 1% HCl solution for 5 min, stained with trypan blue, and placed on glass slides for colonization determination. A total of 150 root segments per treatment were examined under the microscope to determine the proportion of the root length that had been colonized by the AM fungi. The presence or absence of AM fungi in roots from inoculated and uninoculated seedlings was further confirmed by nested PCR using the primer pairs SSUmAf/LSUmAr and SSUmCf/LSUmBr [21]. AM dependency was calculated by determining the ratio of the dry weight of mycorrhizal seedlings to the dry weight of nonmycorrhizal seedlings [22].

### 2.5 Growth index and biomass measurement

At the beginning and end of the last 30-day water treatment, the height was measured by tape. The plant height was recorded as the average amount of growth every day. The fully expanded leaf (near the apex) was used to measure the relative chlorophyll content by a SPAD meter (SPAD-502, Minolta, Tokyo, Japan).

At harvest, seedlings were cut into shoot and root parts, placed at 105°C for 20 min in an oven to destroy the enzymes, and then dried at 80°C to a constant weight to determine the biomass of shoots and roots. The root/shoot ratio was calculated by determining the ratio of root biomass to shoot biomass.

### 2.6 Gas exchange measurement

Within a week before harvest, the net photosynthesis (Pn), stomatal conductance (Gs), intercellular CO_2_ concentration (Ci) and transpiration rate (E) of the fully expanded leaves of six randomly selected seedlings in each treatment were measured at a light intensity of 1,000 μmol (photon) m^-2^ S^-1^ between 08:00 and 11:30 h using a *Li*-*6400* portable photosynthesis measurement system (Li-Cor Inc., Lincoln, NE, USA). The parameters were set as follows: temperature, 30°C; leaf-air vapor pressure deficit, 1.5±0.5 kPa; relative humidity, 50%; and ambient CO_2_, 350 μmol mol^−1^.

### 2.7 Chlorophyll fluorescence measurement

The fully expanded leaves near the apex of six randomly selected plants of each treatment were placed in the dark for 30 min at room temperature before chlorophyll fluorescence measurement. The maximal fluorescence (Fm) and the minimum fluorescence (Fo) yields were determined using a modulated chlorophyll fluorometer (MINI-Imaging-PAM, Walz, Germany). The actual quantum yield of photosystem II (PSII) (ΦPSII), the maximum quantum yield of PSII (Fv/Fm), the nonphotochemical quenching (qN) and the photochemical quenching (qP) were calculated as follows:

Fv/Fm = (Fm−Fo)/Fm;
ΦPSII = (Fm′−F)/Fm′;
qN = 1−(Fm′−Fo′)/(Fm−′Fo);
qP = (Fm′−F)/(Fm′−Fo′).

### 2.8 Antioxidant enzyme activities measurement

In each treatment, six plants were selected randomly to analyze the superoxide dismutase (SOD, EC1.15.1.1), catalase (CAT, EC1.11.1.6) and peroxidase (POD, EC1.11.1.7) activities. The fully expanded leaves near the apex and roots were used for measurement. According to Bradford method, soluble protein concentrations were assayed with Coomassie Brilliant Blue G-250 by the absorbance at 595 nm, and the bovine serum albumin was used as a protein standard [23]. SOD activity was assayed by measuring the superoxide radicals generated photochemically at 560 nm [24]. As described by Mallick and Mohn, CAT and POD activities were assayed by the absorbance at 240 nm and 470 nm [25].

### 2.9 Statistical analysis

All of the data were analyzed using the statistical analysis software SPSS 22.0 (SPSS Inc., Chicago, IL, USA). Two-way analyses of variance (ANOVA) were adapted to evaluate the significance of the effects of a water deficit, AM inoculation and their interaction on seedlings at the significance level of *P* ≤ 0.05. The means were compared using Duncan’s multiple range test and HSD test (*P* ≤ 0.05). Principal component analysis (PCA) was implemented to reduce all the parameters to the fewest dimensions while keeping the eigenvalue >1. Before PCA, the correlation analyses were performed by Pearson correlation coefficients, highly correlated variables were removed (only one remained), and all remaining data were standardized and used for PCA. All figures were made using Origin 9.1.

## 3. Results

### 3.1 Water regimes in the weight loss of pots

In the first 3 months, the pots lost 45–55 g in weight every day, and it took approximately 4 d for the water content in the water deficit treatments to change from 100% to 20% of FC before the water deficit treatment period. During the water deficit treatment period, the average weights lost every day in the 20%, 40%, 60%, 80% and 100% FC treatments were approximately 32, 37, 41, 45 and 50 g, respectively, and showed slight increases with time.

### 3.2 AM inoculation status

**Fig 1:**
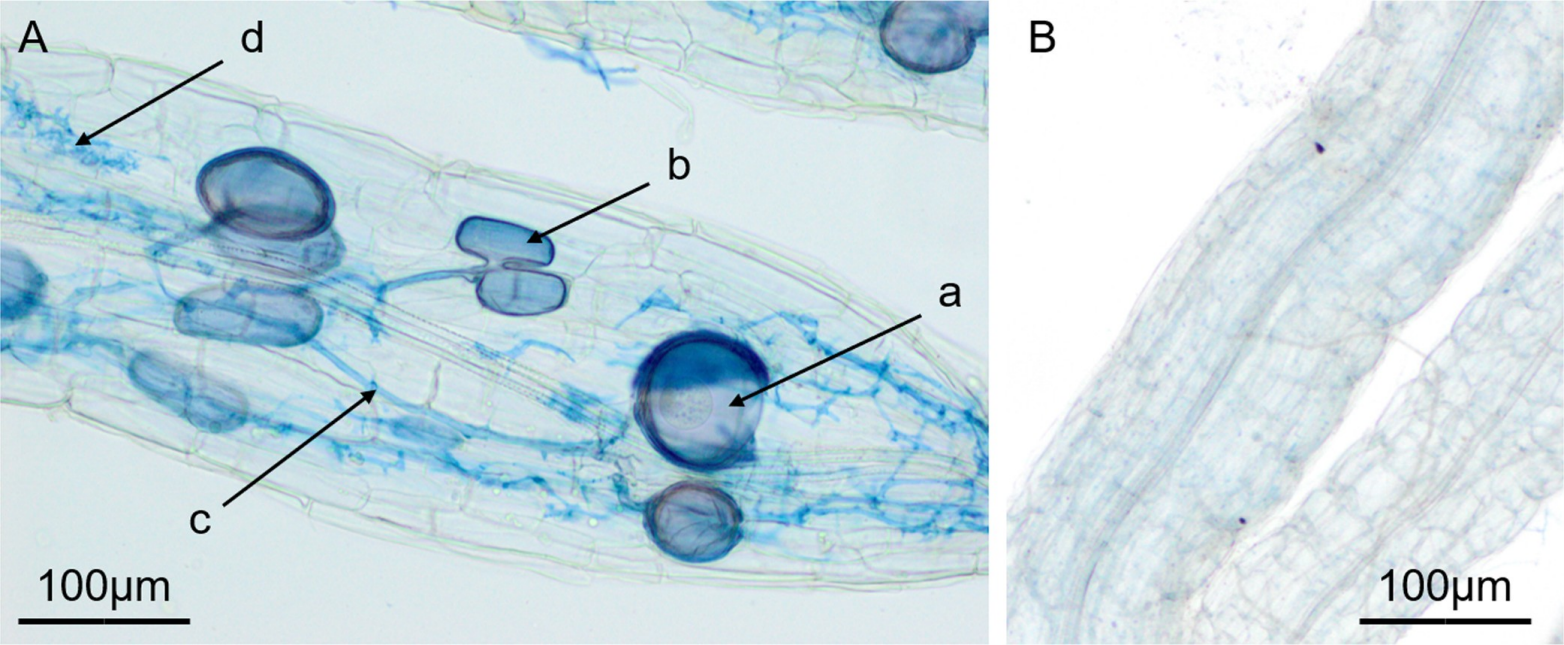
AM structures detected in mycorrhizal root (A) and root of non-mycorrhizal seedlings (B). a: spore; b: vesicle; c: hypha; d: arbuscule.

**Fig 2:**
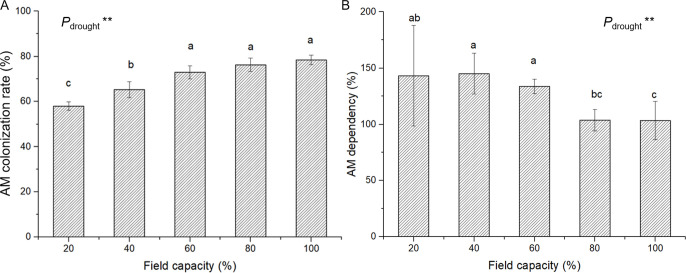
AM colonization rate (A) and AM dependency (B) in *E*. *maackii* under water deficit. AM: AM inoculated treatment; NM: uninoculated treatment; **: *P* ≤ 0.01. Means with a letter in common within each variable can’t be considered different at P ≤ 0.05.

Seedlings that had received AM inoculation treatment formed typical AM structures, and in all nonmycorrhizal treatments, no AM structure was detected in plant roots (Fig 1). The water deficit decreased the AM inoculation rate from 78.40% (100% FC treatment) to 58.00% (20% FC treatment) and improved the AM dependency from 103.46% (100% FC treatment) to 143.13% (20% FC treatment) (Fig 2). There was no significant difference between the AM inoculation rates and AM dependencies of seedlings in the 100%, 80% and 60% FC treatments. ANOVA results indicated significant effects of water deficit levels on the AM inoculation rate and AM dependency (S1 Table).

### 3.3 Growth and biomass accumulation

**Fig 3:**
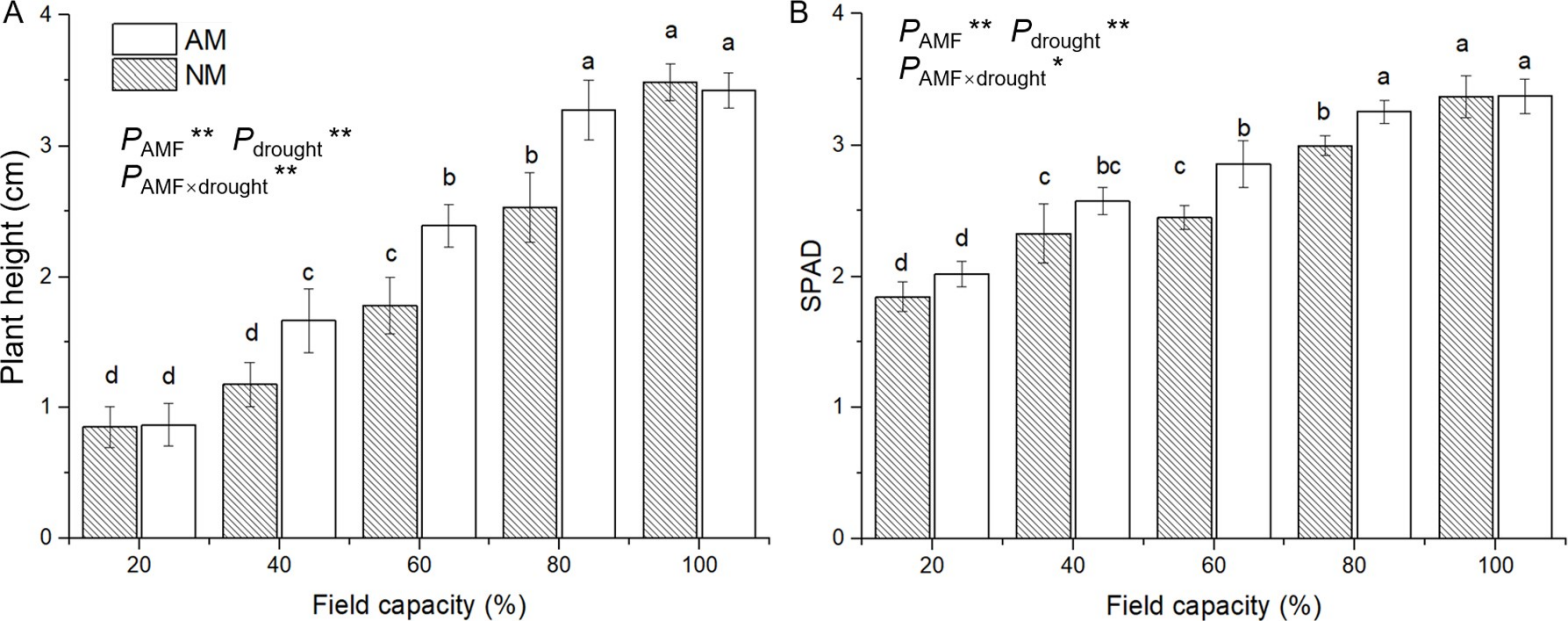
Effect of AM inoculation on plant height (A) and SPAD (B) of *E*. *maackii* under water deficit. AM inoculated treatment; NM: uninoculated treatment; AMF: effect of AM inoculation; drought: effect of water deficit; AMF×drought: effect of interaction of AM inoculation and water deficit treatment; *: *P* ≤ 0.05; **: *P* ≤ 0.01. Means with a letter in common within each variable can’t be considered different at P ≤ 0.05.

Seedlings were affected by water deficit and showed significantly reduced plant height and SPAD compared with seedlings grown at 100% FC (Fig 3). Compared with nonmycorrhizal seedlings, mycorrhizal seedlings showed significantly higher plant height under moderate drought conditions (40%, 60% and 80% FC) and higher SPAD of seedlings in the 60% and 80% FC treatments. Moreover, under extreme drought treatment (20% FC), no obvious difference was detected between mycorrhizal and nonmycorrhizal seedlings with regards to the plant height and SPAD. ANOVA results suggested that the plant height and SPAD were significantly affected by AM inoculation and drought and their interaction (S1 Table).

**Fig 4:**
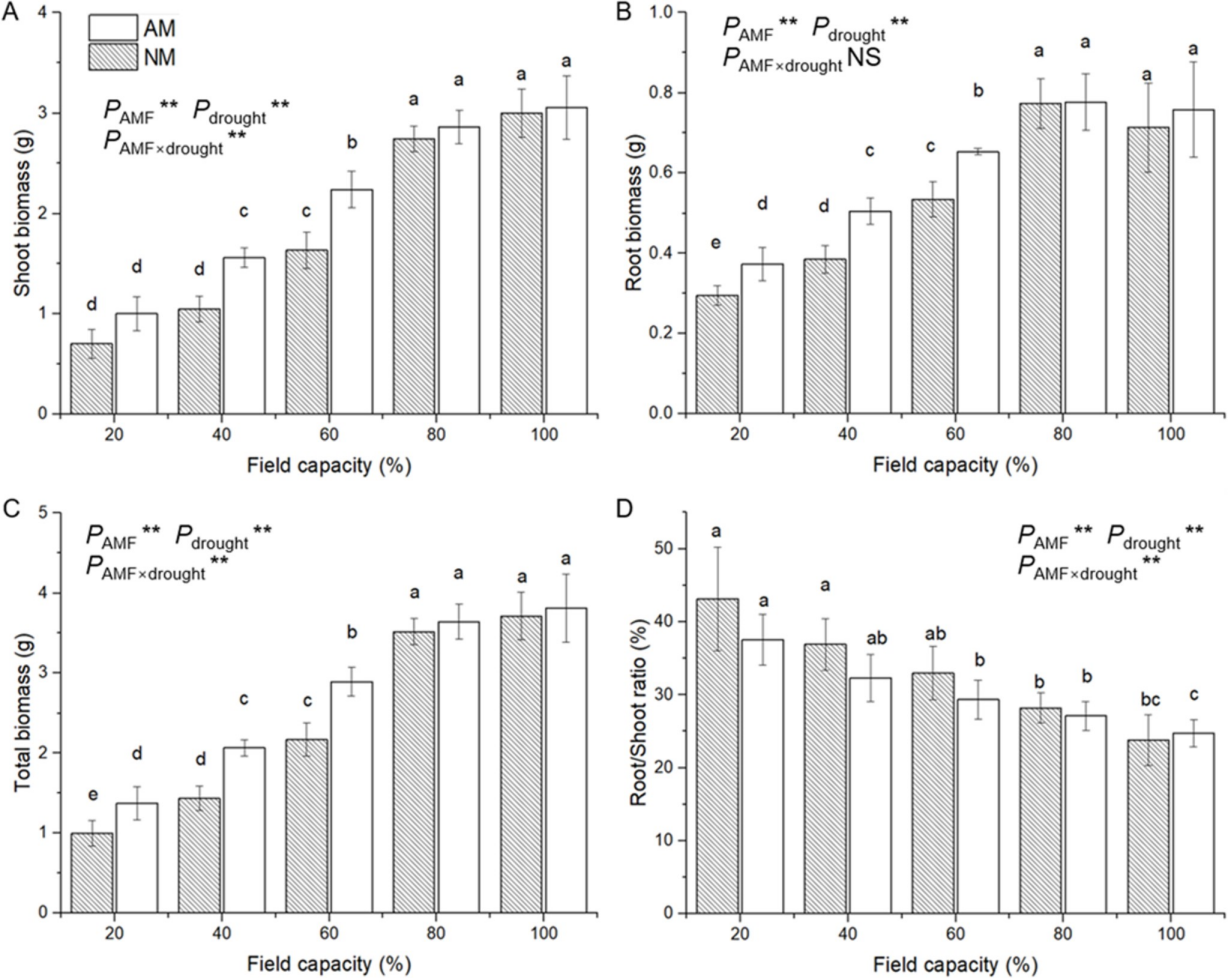
Effect of AM inoculation on biomass of shoot (A), root (B) and total (C) and root/shoot ratio (D) of *E*. *maackii* under water deficit. AM inoculated treatment; NM: uninoculated treatment; AMF: effect of AM inoculation; drought: effect of water deficit; AMF×drought: effect of interaction of AM inoculation and water deficit treatment; NS: no significant effect; **: *P* ≤ 0.01. Means with a letter in common within each variable can’t be considered different at P ≤ 0.05.

Compared with seedlings at 80% and 100% FC, the shoot biomass, root biomass, and total biomass of seedlings that had been subjected to the more serious water deficit treatment (20%, 40% and 60% FC) were significantly lower (Fig 4). The effect of AM inoculation was not obvious in the 80% and 100% FC treatments in the shoot biomass, root biomass, and total biomass. Furthermore, seedlings grown under water deficit conditions of 40% and 60% FC had significantly lower shoot biomass, root biomass, and total biomass than seedlings that had received the inoculation treatment. In addition, a water deficit gradually decreased the root/shoot ratio, whereas the effect of AM inoculation was not significant. ANOVA results suggested a significant effect of AMF and a water deficit on the shoot biomass, root biomass, and total biomass and a significant effect of the interaction of the AMF and water deficit on the shoot biomass, total biomass, and root/shoot ratio (S1 Table).

### 3.4 Gas exchange

**Fig 5:**
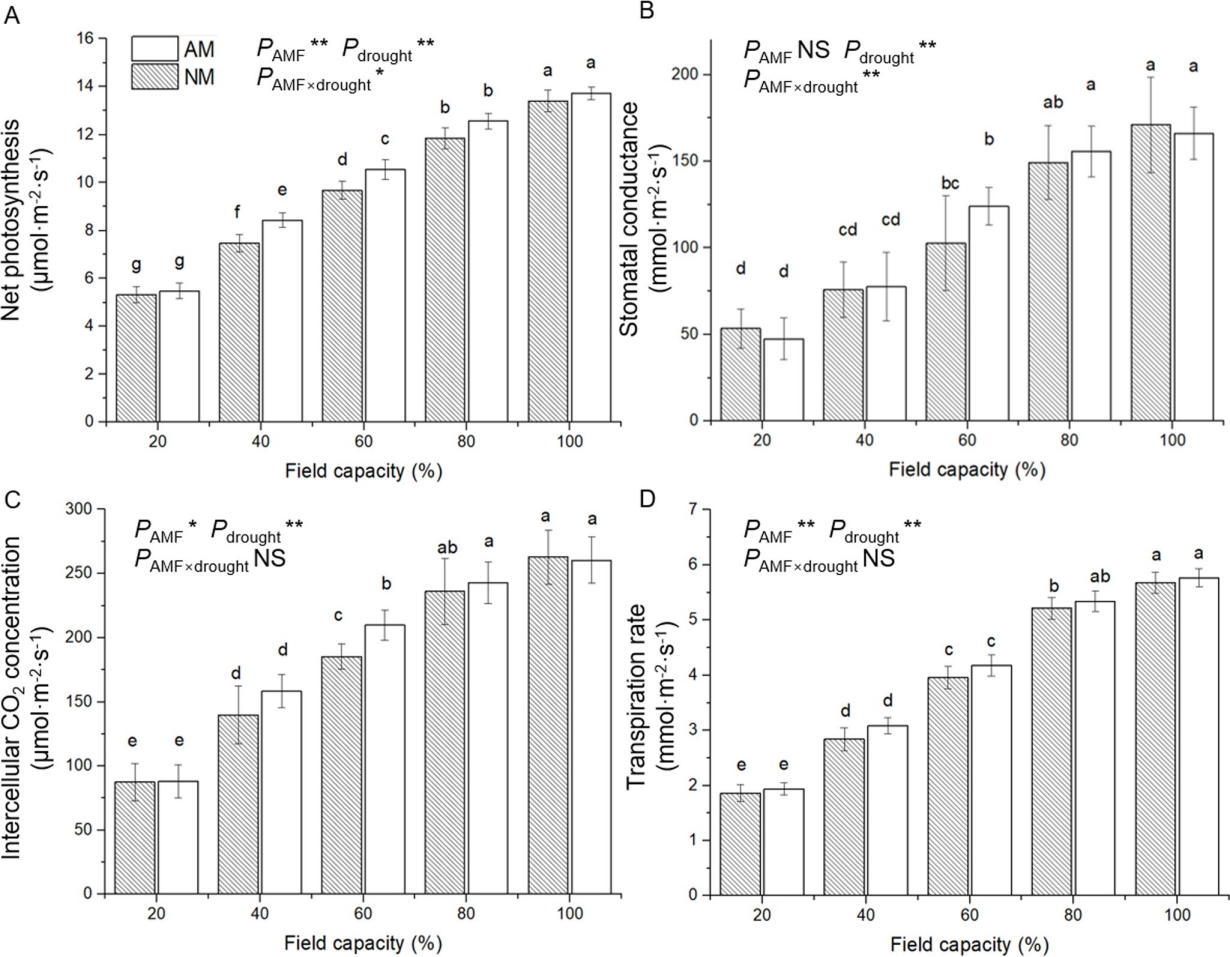
Effect of AM inoculation on gas exchange indexes, Pn (A), Gs (B), Ci (C) and E (D) of *E*. *maackii* under water deficit. AM inoculated treatment; NM: uninoculated treatment; AMF: effect of AM inoculation; drought: effect of water deficit; AMF×drought: effect of interaction of AM inoculation and water deficit treatment; NS: no significant effect; *: *P* ≤ 0.05; **: *P* ≤ 0.01. Means with a letter in common within each variable can’t be considered different at P ≤ 0.05.

The gas exchange measurements recorded for mycorrhizal and nonmycorrhizal seedlings grown under a water deficit were similar: net photosynthesis, stomatal conductance, intercellular CO_2_ concentration and transpiration rate were gradually limited by the gradient water deficit (Fig 5). The inoculation of seedlings with AM fungi had no significant effect on the stomatal conductance and transpiration rate. In the 20%, 80% and 100% FC treatments, no significant difference was detected between mycorrhizal and nonmycorrhizal seedlings in net photosynthesis and intercellular CO_2_ concentration. Meanwhile, compared with nonmycorrhizal seedlings, mycorrhizal seedlings showed significantly higher net photosynthesis and intercellular CO_2_ concentration.

ANOVA results suggested that AM inoculation had a significant effect on the net photosynthesis, intercellular CO_2_ concentration and transpiration rate, and the effect of the water deficit was significant on all gas exchange parameters detected. However, the interaction of AM inoculation and the water deficit only significantly affected net photosynthesis and stomatal conductance (S1 Table).

### 3.5 Chlorophyll fluorescence

**Fig 6:**
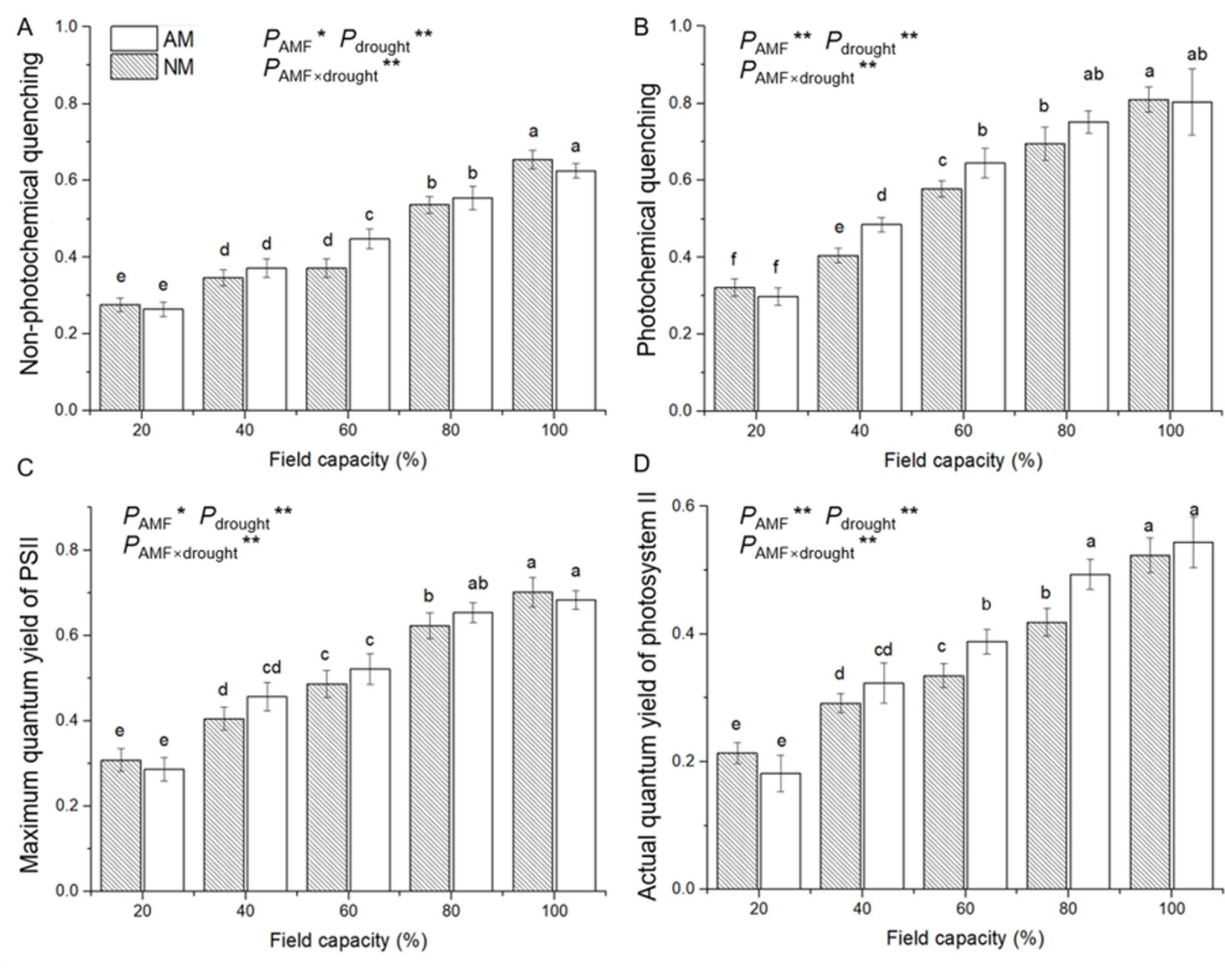
Effect of AM inoculation on chlorophyll fluorescence indexes, qN (A), qP (B), Fv/Fm (C) and ΦPSII (D) of *E*. *maackii* under water deficit. AM inoculated treatment; NM: uninoculated treatment; AMF: effect of AM inoculation; drought: effect of water deficit; AMF×drought: effect of interaction of AM inoculation and water deficit treatment; *: *P* ≤ 0.05; **: *P* ≤ 0.01. Means with a letter in common within each variable can’t be considered different at P ≤ 0.05.

By the end of the water deficit treatment period, seedlings grown under the gradient water deficit showed gradually decreasing qN, qP, Fv/Fm and ΦPSII (Fig 6). The qN among seedlings inoculated with AM fungi was significantly higher than that among uninoculated seedlings under 60% FC. Seedlings that received AM inoculation treatment had significantly higher qP under 40% and 60% FC compared with uninoculated seedlings. Under 40%, 60% and 80% FC conditions, inoculated seedlings showed significantly higher ΦPSII than uninoculated seedlings. However, no significant effect of AM inoculation was detected in the Fv/Fm of seedlings grown under all water deficit conditions. ANOVA results indicated that the effects of AM inoculation, water deficit and their interaction were significant on all of these gas exchange parameters (S1 Table).

### 3.6 Antioxidant enzyme activities

**Fig 7:**
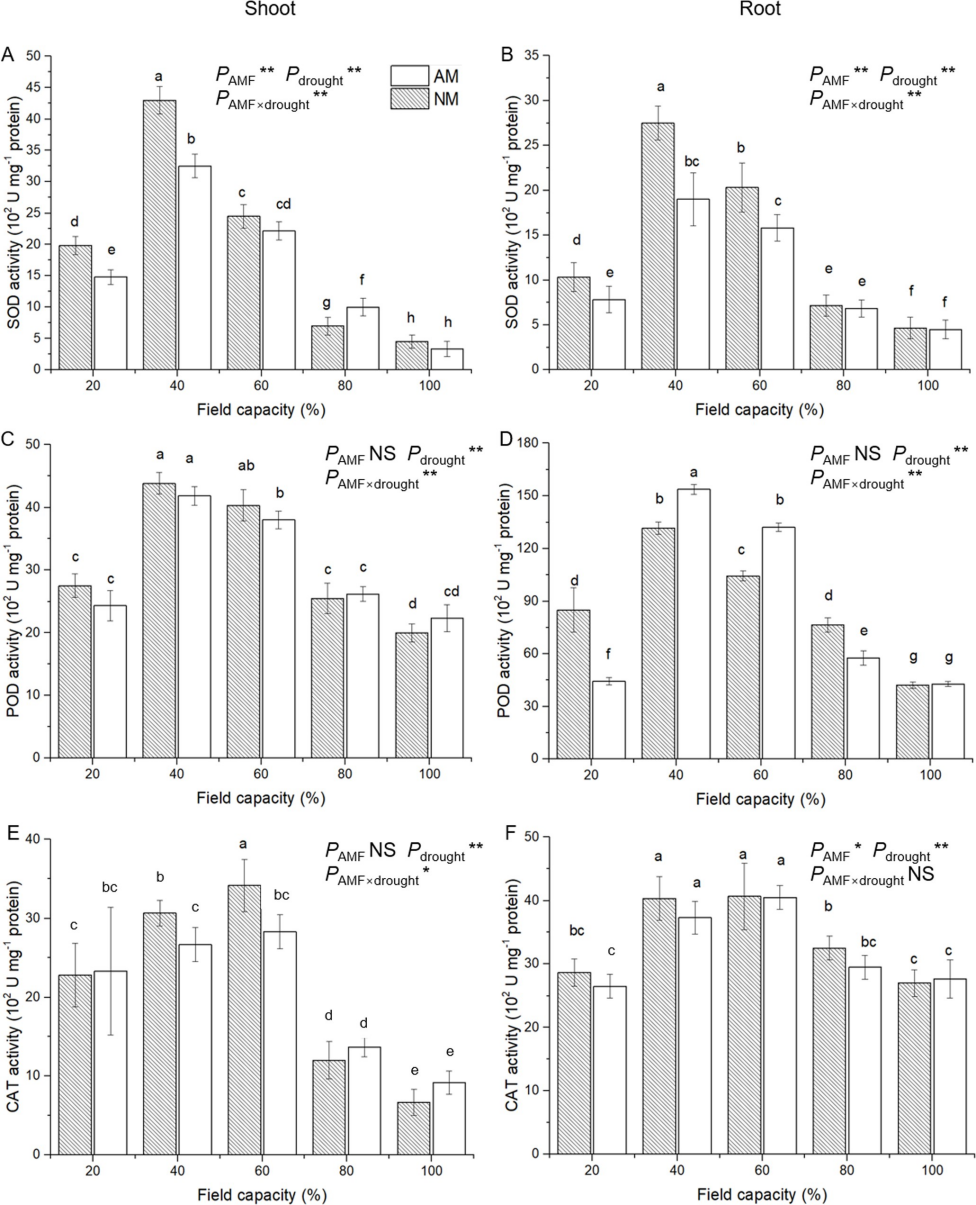
Effect of AM inoculation on SOD (A, B), POD (C, D) and CAT (E, F) activities in shoot and root of *E*. *maackii* under water deficit. AM inoculated treatment; NM: uninoculated treatment; AMF: effect of AM inoculation; drought: effect of water deficit; AMF×drought: effect of interaction of AM inoculation and water deficit treatment; NS: no significant effect; *: *P* ≤ 0.05; **: *P* ≤ 0.01. Means with a letter in common within each variable can’t be considered different at P ≤ 0.05.

Compared with the growth indexes detected above, the trend of changes in the antioxidant enzymatic activities along with water deficit levels was complicated (Fig 7). The SOD, POD and CAT activities of shoots and roots showed similar changes along with the water deficit levels: increased from 100% to 60% FC, peaked under 40% or 60% FC, and decreased under 20% FC. However, SOD, POD and CAT showed different responses to AM inoculation treatment. The SOD activities of the shoot and root among seedlings that received AM inoculation treatment were significantly lower than those of uninoculated seedlings under 20% and 40% FC conditions. In contrast, AM inoculation significantly improved the SOD activity in shoots under 80% FC conditions.

In the shoots, the POD activity of seedlings that received the AM inoculation treatment was not different from that of nonmycorrhizal seedlings. Furthermore, in roots, AM inoculation significantly increased POD activity under 40% and 60% FC conditions, whereas the opposite effect of AM inoculation treatment was detected under 80% and 20% FC conditions. In addition, the intensity of the change in POD activities along with water deficit levels in roots was greater than that in shoots. In shoots, seedlings that received AM inoculation treatment showed significantly lower CAT activity under 40% and 60% FC conditions. For CAT activity in roots, AM inoculation had no effect.

ANOVA results indicated that AM inoculation had a significant effect on the SOD activities in shoots and roots and CAT activity in roots; water deficit treatment had a significant effect on the SOD, POD and CAT activities; and the interaction of AM inoculation and water deficit treatment significantly affected all of the enzymatic activities (except CAT activity in roots) (S1 Table).

### 3.7 PCA results

**Fig 8:**
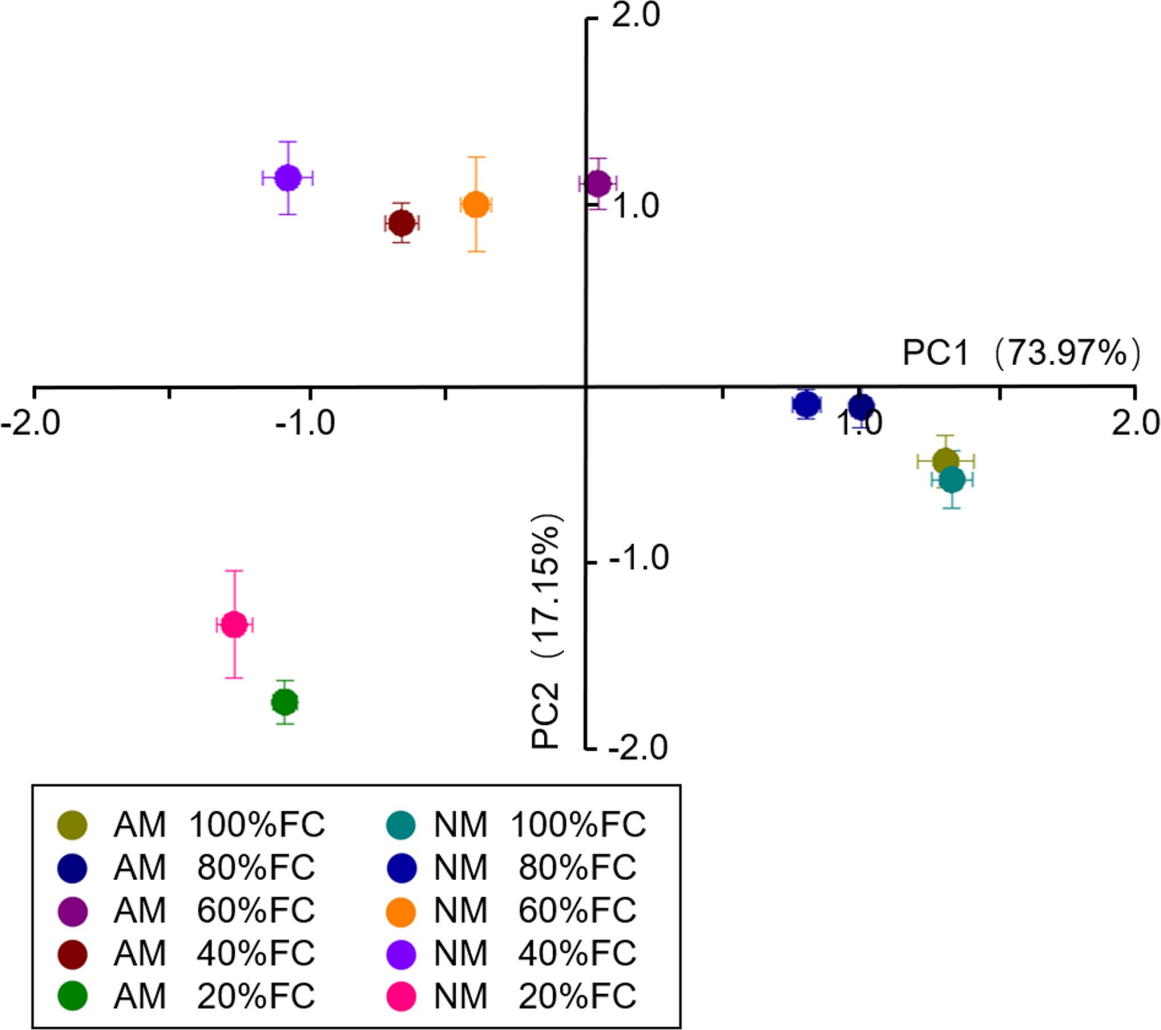
PCA results. AM inoculated treatment; NM: Uninoculated treatment.

To investigate the effect of AM inoculation on the growth, photosynthesis, and antioxidant enzymatic activity of *E*. *maackii* under gradient water deficit levels, PCA was performed using all of the experimental data sets. Before PCA, correlation analysis was calculated, and highly correlated variables were removed. The plant height and CAT activities in roots were selected as presentations for the following PCA process. Among all seedlings, principal component 1 (PC1) and principal component 2 (PC2) accounted for 73.97% and 17.15% of the variance, respectively (Fig 8). PC1 tended to separate the water deficit effects, which suggested a greater effect of the water deficit compared with AM inoculation. In PC1, under 20%, 40%, 60% and 80% FC conditions, seedlings that received AM inoculation treatment were closer to seedlings under the 100% FC condition than nonmycorrhizal seedlings, which meant that AM inoculation had a positive effect on the growth, photosynthesis, and antioxidant enzymatic activity of *E*. *maackii* under gradient water deficit levels.

## 4. Discussion

A water deficit is one of the major limitations on plant growth, and as a result of anthropogenic disturbance and predicted global climate changes, drought conditions are expected to increase in the near future [3, 15, 26]. During the last few decades, a large number of studies have focused on implementing and validating tools and techniques to prevent drought-mediated damage [27, 28]. In this study, we investigated the effect of the AM fungal species *R*. *intraradices* on improving drought tolerance mechanisms in *E*. *maackii* under gradient water deficit conditions.

A water deficit had a negative effect on AM inoculation [4, 13, 15, 29], which supported our findings that AM inoculation rates in seedlings along with an increasing water deficit gradually decreased. The water deficit was the key limitation in plant growth [15, 27, 29, 30]. The improvement in plant growth in the AM inoculation treatment could be attributed to the maintenance of increased mineral availability for absorption and water content in plant tissue [31]. Many researchers have reported the positive role of AM symbiosis in plant growth under drought stress [15, 32]. Among AM-inoculated damask rose, a significant increase in the uptake of essential mineral elements was detected compared with nonmycorrhizal plants under a severe water deficit, reflected in the growth and drought tolerance improvement [33]. Under water deficit conditions, AM fungi could modify the root morphology, such as increasing the root fineness, root/shoot ratio, and length of hair roots, resulting in an improvement in mineral element uptake [34, 35].

Water deficit had a direct negative effect on plant photosynthesis, resulting from greater water absorption pressure and a reduction in coupling factors [36]. Begum [35] suggested that a severe water deficit reduced pigment accumulation and photosynthesis attributes, which were alleviated by AM inoculation. AM-mediated increases in the chlorophyll II content and photosynthesis rate in damask rose under a water deficit were reported [33]. Similar to our findings, improvement in the gas exchange and PSII activity in seedlings that received AM inoculation treatment positively enhanced the photosynthesis function over water deficit and nonmycorrhizal seedlings.

Under moderate water deficit conditions (40%, 60% and 80% FC), seedlings that received AM inoculation treatment showed better performance in gas exchange and chlorophyll fluorescence indexes compared to the nonmycorrhizal treatment. This confirmed the report of Li et al. [15], suggesting the role of AM fungi in substantially increasing the host plant’s drought tolerance. The formation of AM symbiosis could increase the root hydraulic conductivity and stomatal regulation in the host plant, improving contact with soil particles and water extraction, which supplied a better water regime for photosynthesis [37, 38].

As reported, reduced water availability limited the expression of growth-associated genes [39]. Similar to our results, decreased biomass accumulation along with an increased water deficit level was directly regulated by decreased photosynthetic function. In addition, AM inoculation could significantly ameliorate the induced declines in growth and biomass accumulation by a water deficit [40], which supported our findings that AM inoculation alleviated the negative effect of a water deficit in the shoot biomass, rot biomass and total biomass under 20%, 40% and 60% FC conditions. Leaves were more sensitive to drought than roots, which was in line with our results that root/shoot ratio increased slightly with increasing water deficit levels.

Drought mediates an imbalance in reactive oxygen species such as H_2_O_2_ and O_2_^−^, causing severe oxidative damage and upregulating antioxidant enzymatic activities [8, 41]. Similar to our results, the water deficit induced SOD, POD and CAT activities in both shoots and roots to improve from the 100% FC treatment to the 40% FC treatment. However, significant decreases in the SOD, POD and CAT activities were observed under severe water deficit conditions (20% FC), which may be caused by a severe drought limitation. AM inoculation showed limited SOD activities in both shoots and roots under 20%, 40% and 60% FC conditions, which suggested that mycorrhizal seedlings were less sensitive to a water deficit than nonmycorrhizal seedlings [35]. Maintaining lower antioxidant enzymatic activity suggested a lower reactive oxygen species concentration, which benefits seedlings in regulating development processes, such as root growth, signaling, stomatal function and cell senescence [15, 42, 43]. However, the roots of seedlings that had received AM inoculation treatment showed significantly higher POD activity under 40% and 60% FC conditions, which was in line with the research of Begum et al. [35]. Furthermore, there was no significant difference between mycorrhizal and nonmycorrhizal seedlings in CAT activity. The loss of antioxidant enzymatic stability intensified the reduction in photosynthesis, which resulted from upregulating chlorophyllase activity and reducing Rubisco synthesis by environmental stress [44, 45]. The fact that well-watered plants showed the lowest antioxidant enzymatic activities supports the idea that water deficit caused damages in antioxidase system, while mycorrhizal seedling maintaining lower antioxidant enzymatic activity suggested positive role of AM fungi in drought tolerance.

Many studies have suggested better performance of AM fungi under moderate drought stress and ineffective roles under severe drought conditions [46, 47]. A severe water deficit caused the reduction of the effectiveness of AM inoculation in wheat, which in turn resulted in prolonged water stress [29]; this supported our findings that AM inoculation benefited the growth, photosynthesis and antioxidant enzymatic activity of seedlings that had been subjected to a moderate water deficit, especially under 40%, 60% and 80% FC conditions, whereas AM inoculation was ineffective under the 20% FC condition.

## 5. Conclusion

In conclusion, water deficit induced a negative influence on the growth and development of *E*. *maackii*, reflected in limitations in growth and biomass accumulation, photosynthesis decline and antioxidant enzymatic activity improvement. Inoculation with AM fungus (*R*. *intraradice*s) alleviated these negative effects and benefited *E*. *maackii*, especially under moderate water deficit stress (40% and 60% FC), which suggested the potential role of AMF in ecological stability when suffering from drought. Furthermore, other AM fungi species and a consortium of AM fungus should be discussed which could offer more possibilities.

## Supporting information

S1 Table Detail on the statistical analyses. (DOC)

## Abbreviations

AM: arbuscular mycorrhizal
FC: field capacity
PC: principal component
Pn: net photosynthesis
Gs: stomatal conductance
Ci: intercellular CO_2_ concentration
E: transpiration rate
Fm: maximal fluorescence
Fo: minimum fluorescence
PSII: photosystem II
ΦPSII: actual quantum yield of PSII
qN: non-photochemical quenching
qP: photoc hemical quenching
Fv/Fm: maximum quantum yield of PSII
ANOVA: analyses of variance
PCA: principa component analysis
SOD: superoxide dismutase
CAT: catalase
POD: peroxidase
