# Supplementary material for: Effects of arbuscular mycorrhizal inoculation on the growth, photosynthesis and antioxidant enzymatic activity of Euonymus maackii Rupr. under gradient water deficit levels
Source: PLoS One. 2021 Nov 23;16(11):e0259959. doi: 10.1371/journal.pone.0259959 (PMC8610274; doi:10.1371/journal.pone.0259959)
Supplement: S1 Table — (DOC) [file pone.0259959.s001.doc]

S1 Table. Detail on the statistical analyses


Growth indexes	AM inoculation	Drought	AM inoculation ×Drought	
	P value	F value	DF	P value	F value	DF	P value	F value	DF	
Plant height	0.000	51.980	1	0.000	362.253	4	0.000	10.658	4	
BS	0.000	44.373	1	0.000	307.313	4	0.002	5.001	4	
BR	0.000	19.109	1	0.000	101.209	4	0.148	1.780	4	
TB	0.000	43.836	1	0.000	288.207	4	0.003	4.646	4	
Root/Shoot ratio	0.004	8.975	1	0.000	35.958	4	0.172	1.668	4	
SPAD	0.000	40.593	1	0.000	218.104	4	0.010	3.715	4	
Pn	0.000	41.352	1	0.000	969.200	4	0.036	2.794	4	
Gs	0.087	3.039	1	0.000	440.157	4	0.001	5.440	4	
Ci	0.044	4.261	1	0.000	179.506	4	0.318	1.211	4	
E	0.002	11.214	1	0.000	970.669	4	0.670	0.592	4	
WUEi	0.056	3.813	1	0.000	64.358	4	0.523	0.812	4	
qN	0.012	6.805	1	0.000	483.098	4	0.000	9.216	4	
qP	0.001	12.235	1	0.000	334.034	4	0.004	4.396	4	
FvFm	0.048	4.120	1	0.000	354.665	4	0.008	3.877	4	
PSII	0.000	20.790	1	0.000	320.175	4	0.000	7.647	4	
SOD activity of shoot	0.000	63.823	1	0.000	881.804	4	0.000	31.118	4	
SOD activity of root	0.000	48.505	1	0.000	238.058	4	0.000	11.295	4	
POD activity of shoot	0.075	3.311	1	0.000	290.967	4	0.004	4.355	4	
POD activity of root	0.159	2.048	1	0.000	878.642	4	0.000	102.748	4	
CAT activity of shoot	0.247	1.375	1	0.000	104.481	4	0.014	3.474	4	
CAT activity of root	0.039	4.514	1	0.000	60.268	4	0.369	1.095	4	


Note: DF, degree of freedom.
